# West Nile and Usutu Virus Introduction via Migratory Birds: A Retrospective Analysis in Italy

**DOI:** 10.3390/v14020416

**Published:** 2022-02-17

**Authors:** Elisa Mancuso, Jacopo Giuseppe Cecere, Federica Iapaolo, Annapia Di Gennaro, Massimo Sacchi, Giovanni Savini, Fernando Spina, Federica Monaco

**Affiliations:** 1 Istituto Zooprofilattico Sperimentale dell’Abruzzo e del Molise ‘G. Caporale’, 64100 Teramo, Italy; f.iapaolo@izs.it (F.I.); a.digennaro@izs.it (A.D.G.); g.savini@izs.it (G.S.); f.monaco@izs.it (F.M.); 2 Area Avifauna Migratrice, Istituto Superiore per la Protezione e la Ricerca Ambientale (ISPRA), Via Ca’ Fornacetta, Ozzano dell’Emilia, 40064 Bologna, Italy; jacopo.cecere@isprambiente.it (J.G.C.); massimo.sacchi@isprambiente.it (M.S.); fernando.spina@isprambiente.it (F.S.)

**Keywords:** West Nile virus, Usutu virus, flavivirus, zoonoses, migratory birds, Italy

## Abstract

The actual contribution of migratory birds in spreading West Nile (WNV) and Usutu virus (USUV) across Europe and from Africa to old countries is still controversial. In this study, we reported the results of molecular and serological surveys on migrating birds sampled during peaks of spring and autumn migration at 11 Italian sites located along important flyways, from 2012 to 2014. A total of 1335 specimens made of individual or pooled sera, and organs from 275 dead birds were tested for WNV and USUV RNA by real time PCR (RT-PCR). Furthermore, sera were tested by serum neutralization assay for detecting WNV and USUV neutralizing antibodies. Molecular tests detected WNV lineage 2 RNA in a pool made of three Song Thrush (*Turdus philomelos*) sera sampled in autumn, and lineage 1 in kidneys of six trans-Saharan birds sampled in spring. Neutralizing antibodies against WNV and USUV were found in 5.80% (*n* = 72; 17 bird species) and 0.32% (*n* = 4; 4 bird species) of the tested sera, respectively. Our results do not exclude the role of migratory birds as potential spreaders of WNV and USUV from Africa and Central Europe to Mediterranean areas and highlight the importance of a more extensive active surveillance of zoonotic viruses.

## 1. Introduction

Among terrestrial vertebrates, birds have the physical ability to move through continents for thousands of kilometers in a few days, crossing ecological barriers, such as mountains, deserts and seas. Migratory avian species of the Western Palearctic (Europe, Middle East and Northern Africa) have developed countless seasonal North-South routes represented either by movements within the region itself (short-distance or intra-Palearctic migrants) or by reaching sub-Saharan Africa (long-distance or trans-Saharan migrants). These birds leave the breeding sites and move southward in the late boreal summer/autumn for then flying back northwards in spring for the following breeding season. Migratory behavior can be different among species. It can always be present and genetically determined (obligate migrants) or even be completely absent (resident species). Even within the same species there could be resident and migratory individuals (partial migration). In some species, finally, the migratory behavior is context dependent and it is just triggered by specific habitat conditions at the breeding grounds (irregular migrants). During their journey, migratory birds can vehicle and spread zoonotic pathogens, including viruses, and they are thought to be responsible for the wide geographic distribution of some important arboviruses. West Nile and Usutu viruses are African mosquito-borne viruses, belonging to the *Flavivirus* genus, *Flaviviridae* family, within the Japanese encephalitis virus serocomplex. They use birds as amplifying hosts and mosquitoes as vectors, particularly those belonging to the *Culex* genus. In Europe, their circulation is mainly associated to a sylvatic cycle involving wild birds and ornithophilic mosquitoes, and to an urban cycle based on synanthropic or domestic birds and species of mosquitoes capable of feeding on both birds and mammals [1]. Humans, horses and less frequently other mammals, such as rodents [2] and bats [3], are incidental dead-end hosts. They can be infected but due to the low levels of viremia they may develop, they cannot transmit the infection to vectors [4]. Thus, migratory birds are thought to play a crucial role in first introductions, whereas resident species are mainly involved in the amplification and local circulation of the viruses [5].

West Nile virus (WNV) is nowadays the most widespread virus among the flaviviruses reported in almost all continents. Detected for the first time in Europe in 1958, during the last two decades its presence has been confirmed in several European and Mediterranean countries [6,7,8,9,10,11] becoming endemic in some of them. Major peaks of infection have actually been recorded in Romania [12], Greece [13], and most recently Italy, Serbia and Greece [14]. The genomes of the circulating strains cluster in nine different lineages [9]; among them, lineage 1 (L1) and 2 (L2) are the most frequently reported so far and those responsible for severe diseases in humans, horses and birds [15,16,17,18,19]. Lineage 1 includes strains circulating in Europe, North America, Africa and Australia, whereas lineage 2, previously confined to sub-Saharan Africa, is nowadays the most widespread in the European region [20,21]. In Italy, WNV was first detected in 1998. After ten years of silence, it re-emerged and since then it has been circulating across the country affecting resident birds, horses, humans and mosquitoes [22]. Up to 2011, the viral circulation was due to strains belonging to L1, hereafter the epidemiological scenario increased its complexity when Eastern European L2 strains were detected becoming the predominant lineage circulating in the following years [23,24,25].

Usutu virus (USUV) was originally isolated in South Africa and detected for the first time in Europe in 2001 where it caused severe mortality in the Austrian Blackbird populations [26,27]. In the following years, USUV spread to Central and Eastern Europe [28,29,30,31,32] affecting wild birds including populations from Italy where the virus has probably been circulating since 1996 [33]. USUV is considered to have a mild zoonotic potential, and infections in humans are usually asymptomatic. Nevertheless, after the first case of neuroinvasive illness associated with USUV infection described in humans in 2009 [34,35], an increasing number of cases mainly in immunocompromised patients are now reported [36,37,38]. Apart from the direct consequences caused by USUV and WNV infections in humans, both viruses may represent a risk for safety in the case of asymptomatic donors donating infectious blood or organs. If transfused, in fact, they may be responsible for a serious disease in the recipients [39,40,41]. After decades of study and experimental trials, the role of wild and domestic birds as reservoirs of the WNV and USUV has been partly elucidated confirming the high susceptibility of some avian orders [42,43,44,45] and proving their capability to migrate while infectious [46].

We report the results of three-year sampling on birds captured at stop-over sites in Italy while flying from Africa in spring and from Central/East Europe during fall migrations. The aim of this large-scale investigation on migratory birds was to provide evidences by using molecular and serologic methods of their role in introducing West Nile and Usutu viruses in Italy during the three-year (2012, 2013 and 2014) study period.

## 2. Materials and Methods

### 2.1. Study Areas and Bird Trapping Techniques

**Figure 1:**
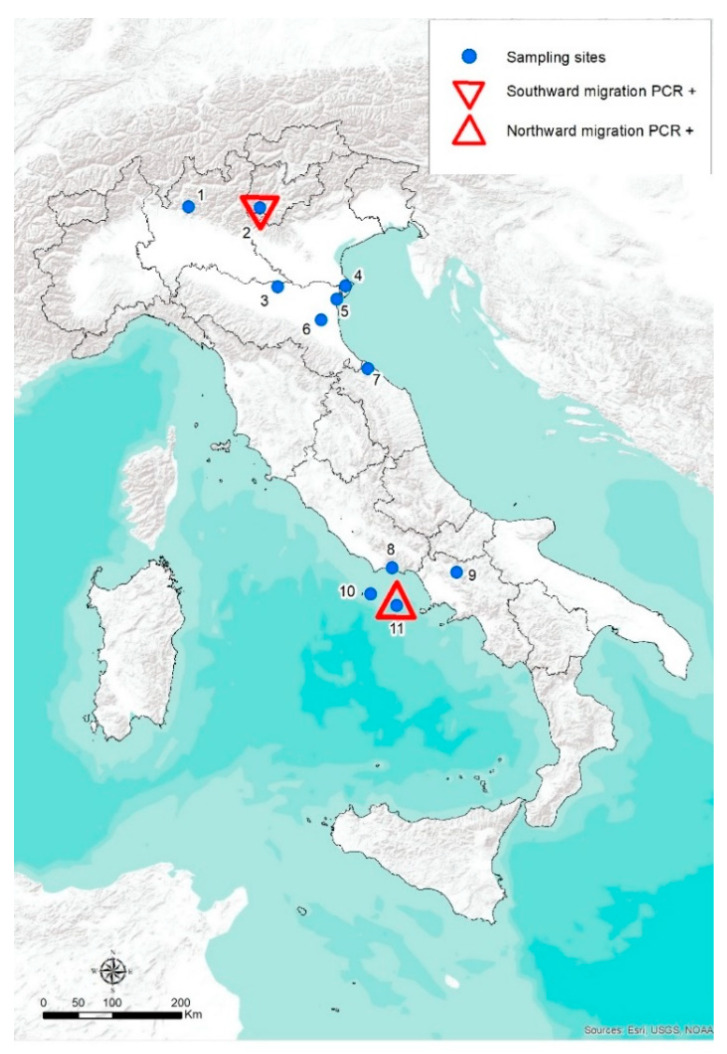
Bird-ringing sites selected in the study. The triangles show the sites where WNV RT-PCR positive birds were found (1 = Barro Mountain, 2 = Bocca di Caset, 3 = Mirandola, 4 = Comacchio Salina, 5 = Ortazzo, 6 = Campotto, 7 = Brisighella Mountain, 8 = Alfieri Lake, 9 = Matese Lake, 10 = Zannone Island, 11 = Ventotene Island).

The study was carried out during a three-year period, from fall 2012 to fall 2014. Migratory birds were caught and sampled during ringing activities at some stopover sites located along the migration routes followed across the Italian territory. Stations with the highest capture rates and number of species per season were chosen to provide the most abundant and heterogeneous samples. All personnel manipulating wild birds was authorized by local administrators and by the Italian Institute for Environmental Protection and Research (ISPRA). The investigation was focused on the avian species belonging to the orders considered highly competent for flaviviruses infection, such as Passeriformes and Charadriiformes [47], but also nocturnal and diurnal raptors (Strigiformes, Falconiformes and Accipitriformes); birds belonging to other non-passerine orders were sampled only if they were occasionally trapped. Overall, 11 different ringing sites were included (Figure 1): 2 in the Alpine region along the main fall migration corridors (Barro Mountain, Bocca di Caset), 6 in the wetland areas (Comacchio Salina, Ortazzo, Mirandola, Campotto, Alfieri Lake, Matese Lake) used mainly by Charadriiformes during both spring and fall migration, 1 site was located on the east coasts of the Italian peninsula (Brisighella Mountain) and 2 on small islands of the Thyrrenian Sea (Ventotene and Zannone Islands). These islands are among the most important stop-over sites for passerines in the Mediterranean during spring movements, featuring a very intensive migratory flow with ensuing high concentrations of individuals and species. Trapping efforts were influenced by weather conditions and logistical factors, yet operations occurred during periods of peak migration: from March to May (northward migration) and from August to November (southward migration).

### 2.2. Sampling Protocols

Once captured, birds were identified by species, marked with metal rings, measured, sexed and aged where possible, weighed and then bled before being released. Blood was collected from the brachial vein, in non-heparinized microhematocrit capillary glass tubes and dispensed in 1.5 mL tubes using a pipette bulb aid. The volume of blood taken from a single individual varied depending on the size of the bird, but never exceeded 1% of the total body mass (bm) [43]. Thus, blood from individuals heavier than 50 g (bm) was processed individually while samples from smaller individuals (10–50 g bm) were pooled, according to the species, up to 10 birds/pool to reach the minimum volume of blood (500 µL) and, consequently, of serum needed to perform both molecular and serological tests (300 µL in total). Individuals lighter than 10 g were not bled. Once collected, the blood was kept at +4 °C, then centrifuged at 2000× *g* for 10 min and the serum harvested and stored at −20 °C until analyzed.

Furthermore, brain and kidneys were collected from net casualties (due to predation or starvation) during the spring campaigns in 2013 and 2014 in the islands of Ventotene and Zannone. The presence of viral RNAs was assessed by molecular analysis. Organs were excised and collected in cryotubes, then kept in a liquid nitrogen portable tank and stored at −80 °C once in the laboratory.

### 2.3. Molecular and Serological Tests

Total RNA was extracted from all the sera and the supernatant of organs previously homogenized [10] using the BioSprint 96 One for All vet kit and the BioSprint 96 workstation (Qiagen, Hilden, Germany). RNA amplification and detection were performed with a one-step RT-PCR method, using the 7900HT Fast Real-time PCR System both for USUV [27,48,49] and for detection and differentiation of West Nile L1 and L2, according to the protocol of Del Amo and colleagues [50]. Cycle threshold (Ct) values below 45 were considered as positive. Once the molecular assays were completed, the remaining volume, if any, was tested by serum neutralization test (SN) in microtiter plates for both West Nile and Usutu virus neutralizing antibodies according to the World Animal Health (OIE) Manual of Diagnostic Tests 2021 [51,52]. The serum samples were diluted starting from dilution titer 1:5 to 1:640, and an equal volume (100 µL) of 100 TCID_50_ (tissue culture infectious dose) of reference WNV and USUV field strains were added to each dilution. In both, WNV and USUV SN assays, positive control sera of either viruses were included to assess possible cross-reactions. After 1 h at 37 °C and 5% CO_2_ in a humidified incubator, 100 µL of 10^5^ Vero cells were added to each well. The plates were incubated at 37 °C for 5 d. Starting from the third day after incubation, the plates were checked for cytopathic effect (CPE). The positive threshold was set at the 1:10 dilution. The sample was considered positive when it showed more than 90% CPE neutralization at the lowest dilution (1:10). WNV and the USUV may cross react but the detection of neutralizing antibody titers at least 4-fold higher for a given flavivirus over the other(s) is considered a proof of specificity [53]. The positive threshold was set at the 1:10 dilution. The limited amounts of sera precluded any ELISA testing.

### 2.4. Statistical Analyses

Possible differences between the WNV and USUV prevalence found in birds according to orders or migratory season (spring vs. autumn) have been analyzed by using a Chi-square test at a significance level of 0.01.

## 3. Results

### 3.1. Samples and Species Composition

During the 3-year period, 3188 birds belonging to 52 species and 14 different orders were ringed and bled, leading to the collection of 1335 serum specimens. Of the 52 bird species, 39 were long-distance, 3 short-distance, 9 partial and 1 resident/irregular migratory species. When distributed according to the migratory season, 823 specimens from 25 species were sampled during the spring activities while 512 samples from 35 species were collected during the autumn fieldwork sessions. Passeriformes were the most abundant among the collected individuals (66.69%; *n* = 2126) but because of their light body mass, the samples were pooled and thus the number of samples tested was 521 (39.02%). Detailed information about serum collection is reported in Supplementary Materials (Table S1).

During necropsies, 529 organs (269 kidneys and 260 brains) were collected from 275 dead birds belonging to 30 different species of Passeriformes (Table S2, Supplementary Materials), 22 of which were long-distance migrants, 5 short-distance migrants, 1 partial migrant and 2 resident species. The presence of WNV and USUV RNA was investigated in all collected sera (1335 samples) and organs (*n* = 529), while the serological survey was limited to the serum specimens collected between 2013 and 2014 (*n* = 1241 from 44 species).

### 3.2. Molecular and Serological Tests

Real Time RT-PCR was able to identify the WNV lineage 2 genome in a pool of 3 Song Thrush (*Turdus philomelos*) sera collected during the 2014 fall migration in Bocca Caset, northern Italy (Figure 1). The specimen was negative to the serological test.

The presence of WNV RNA lineage 1 was detected in the kidneys of 6 birds belonging to 4 different trans-Saharan Passeriformes species: Icterine Warbler (*Hippolais icterina*) (*n* = 1), Common Whitethroat (*Sylvia communis*) (*n* = 2), Wood Warbler (*Phylloscopus sibilatrix*) (*n* = 1), Pied Flycatcher (*Ficedula hypoleuca*) (*n* = 2). Except for the Icterine Warbler collected in 2013, the positive carcasses were collected in 2014 on the island of Ventotene (Figure 1).

Usutu virus genome was never detected in any of the samples collected in the three-year period.

Of the 1241 sera tested by WNV SN, 72 (5.80%) resulted positive (Table 1). In particular, during spring migration, WNV antibodies were detected in 62 of the 823 sera collected (7.53%, 95% CI 5.92–9.54). The positive samples belonged to 12 species out of the 25 captured (48%). Except for the Eurasian Sparrowhawk (*Accipiter nisus*), spring specimens showing neutralizing antibodies against WNV were all trans-Saharan migrants. European Turtle Dove (*Streptopelia turtur*) (*n* = 24/173; 13.87%) and Eurasian Golden Oriole (*Oriolus oriolus*) (*n* = 15/113; 13.27%) were the species in which WNV antibodies were more frequently detected. Together with the Common Quail (*Coturnix coturnix*), they were the species most commonly tested, representing almost 50% of the serum samples collected in spring. The three species are long-distance migrants, their samples were mostly tested individually due to the large body size (>50 g). During the autumn migration, 418 serum samples belonging to 27 species were tested. WNV antibodies were detected in 10 samples (2.39%, CI 95% 1.32–4.35). These belonged to 5 species (11%) represented by 4 long-distance and 1 short-distance migrant, respectively.

USUV neutralizing antibodies were detected in bird samples collected in 2013 only (Table 1). In spring, 3 samples (0.36%, 95% CI 0.13–1.06) collected from 3 species (16%) resulted positive, while in autumn only 1 sample (0.24%, 95% CI 0.06–1.32) showed antibodies against USUV. All three USUV reactive sera collected during the spring migration were from trans-Saharan migrants, whereas the positive serum from the autumn migration belonged to a partial migrant species.

Contemporaneous low levels of neutralizing antibodies against both viruses (1:10) were observed in three samples from Garden Warbler (*Sylvia borin*), Common Quail and European Turtle Dove. Being impossible to exclude any cross reaction between the viruses, the samples were considered inconclusive. In two pool samples, one from Common Whitethroats and the other from Garden Warblers, different neutralizing antibodies against WNV and USUV were also found. In these cases, the lowest titer was regarded as a result of a cross-reaction and the samples were counted as positives only against the virus with the highest antibody titer (Table 1).

### 3.3. Statistical Analyses

The chi-squared test revealed that the WNV prevalence found in birds migrating during the spring migratory seasons was significantly higher (*p* < 0.01) than that found in birds captured during the autumn migratory seasons. Other statistical analyses could not be performed due to the exiguous number of positive samples.

## 4. Discussion

To investigate the role of migratory birds in spreading WNV and USUV, we collected and tested sera and organs of wild individuals captured or found dead during their migration across Italy from Africa (spring) and from Central-Northern Europe (autumn).

In this study, WNV lineage 2 (L2) RNA was detected in a pooled sample derived from three Song Thrushes captured in the Trentino Region while crossing the Alps following the NE-SW direction in October 2014. The question might arise as to whether these birds were infected in or outside Italy. The phenology of the Song Thrush, supported by rings’ recovery data, clearly indicates that these birds entered Italy crossing the Alpine arch following the NE/SW route [54,55]. According to the migratory direction while flying through the Trentino Region, which is WNV naïve area, it can be inferred that they did not stop in the Italian WNV endemic areas before being caught and that, consequently, it is likely they were infected outside Italy.

Historically, African countries have been claimed as the source of WNV introduction in the European and Mediterranean regions [8]. Migratory birds, which may be infected while in their African non-breeding grounds, were pointed as carriers of the virus to European sites during their northbound spring migration. In the recent past, this dogma has been completely revised according to the evolution of the WNV circulating strains which clearly shows the limited involvement of African strains in the WNV circulation within Europe. In fact, following the first European outbreaks in the 1960s [56,57], WNV has been observed in many European countries, such as Romania (1996), Czech Republic (1997), France (2000, 2003, 2004, 2006), Italy (1998, 2008, 2009), Hungary (2000–2009), Spain and Portugal (2004). In 2010, the eco-climatic conditions in Central European and Mediterranean countries promoted an intensive viral circulation resulted in the transmission of WNV to humans [58]. Outbreaks were reported in Greece, Romania, Hungary, Italy, Portugal, Spain, Russia, and EU neighboring countries, such as Israel and Turkey, becoming, as a matter of fact, the most widely spread arbovirus in the old continent [11,59]. Then, since 2018 with the first detection of WNV lineage 2 in Germany, this virus has been spreading towards northern Europe to the Netherlands in 2020 [60] and again in southern France in 2018 [61] and Spain in 2020 [62]. From an Italian perspective, this implies an increased risk of introduction of WNV in the country with the potential exposure to novel viral influxes twice a year, in spring from southern areas, as claimed in the past, and from Central and North-Eastern European countries in autumn. The existence of a dispersal pathway responsible for the introduction of novel WNV strains from Central/Eastern Europe has been demonstrated on the basis of the genetic similarities between the WNV L2 strains circulating in Italy and those circulating in Europe [24,63].

Although it does not necessarily imply the presence of infectious virus, the detection of the WNV genome in the sample of the Song Thrush appears to reinforce what was previously observed. Data from WNV experimental infection have shown 5–7 days of high level of viremia in Passerines [43]. This is a time-frame which would allow birds to reach Italy while hosting infectious virus in their blood. The WNV “northern” route deserves, in our opinion, a careful evaluation in terms of risk of introduction since the birds crossing the Alps and reaching Italy remained in Central/North Europe during the seasonal peak of WNV circulation [54,64].

The presence of WNV RNA in kidneys of six birds belonging to trans-Saharan species found dead in Ventotene island during spring, suggests a possible remnant of a recent infection. In fact, kidney is, among others, the organ in which WNV has been found most abundant and persistent (up to 6 weeks) [44,65,66,67]. It is likely that these birds became infected while in Central/Southern Africa since this island is one of the first stop-over sites reached in a few hours after leaving Northern African coasts and crossing the Mediterranean Sea. However, due to the geographically huge non-breeding areas for the species which might cover most of sub-Saharan Africa, it is difficult to exactly assess the place where the infection originated. As the infected bird species, while on migration, might spend some time in Northern Africa [54], in stop-over sites where WNV has been previously detected [68,69], the possibility of these birds to get infected with WNV while they were there cannot be discarded [70].

A broad spectrum of different bird species, mainly belonging to Passeriformes and Columbiformes, showed WNV neutralizing antibodies. Indeed, the size of most Passeriformes is smaller than that of birds belonging to the other orders. As a consequence, the number of tested specimens were much lower than the number of sampled animals. With regard to this order, pooling can have influenced the results: in fact, if it is true that a pooled sample represents more individuals and thus increases the probability to include at least a seropositive bird, it is also true that the higher the number of birds per pool, the greater is the dilution effect of the antibody titer if present in few birds only. This however was a necessary compromise to maximize the number of sera collected and to provide a descriptive assessment of WNV and USUV in migratory birds reaching Italian sites.

WNV neutralizing antibodies in birds sampled during spring were detected in 12 out of 22 species. Eleven of them were long-distance migrants reaching Europe after leaving non-breeding grounds in sub-Saharan Africa. Overall, titers varied from 1:10 to 1:320, but titers ≥ 1:80 were detected only in six samples from four species: European Turtle Dove, Eurasian Golden Oriole, Garden Warbler and Sparrowhawk. The latter species, as well as other closely related species such as Goshawks (*Accipiter gentilis*), is regarded as particularly susceptible to WNV infection as noticed in 2004 and 2005 in Hungary and in 2017 in Czech Republic, where many birds died due to severe encephalitis [16,71]. In addition to vectors, birds of prey can be infected per os by consuming infected prey, such as mice or small birds [42,45,72,73]. Furthermore, as larger species live longer and have a larger body surface, the probability of becoming infected and transmitting WNV to a vector is considered relevant [74]. Being a short-distance migratory species spending non-breeding periods mainly in southern Europe, these species could play an important role for the circulation of the virus among European countries.

High neutralizing titers were also detected in European Turtle Dove (1:80 and 1:320) which is known to be a long-distance migrant susceptible to WNV [75] and able to transfer infectious virus while on migration [76]. High antibody titers were detected in Eurasian Golden Oriole (1:80 and 1:320) and Garden Warbler (1:160). Although both species belong to the Passeriformes, Garden Warblers are considerably smaller and the positive results were from pooled samples composed by a higher number of individuals (Table 1). The finding of high SN titer in a pooled sample composed of eight individuals might imply the presence of at least one bird with a high antibody titer or alternatively the presence of several seropositive animals in the same pool. Garden Warbler resulted the most WNV infected migrant species in a serological survey conducted in Spain during spring 2004 [76]. However, among the WNV seropositive species included in this study, Garden Warblers is the species with the smallest breeding populations in Italy and thus the probability of being involved in virus transmission is likely low.

Serological results confirmed that migratory birds, at least some of them, have been exposed to WNV and USUV infection during their lifetimes. In this survey, it appears that the WNV exposure was higher in migratory birds coming from sub-Saharan Africa compared to those from Mediterranean countries, confirming what has been already reported by previous similar surveys [77,78]. However, it is not possible to exactly establish where and when they have been infected. Data on the persistence of WNV neutralizing antibodies in avian species are in fact inconsistent. Some authors affirmed that seropositivity can last for more than one year [45,79] while others sustained a rapid reduction in titers after the exposure to the virus [42,80]. The same is true for the data available on the persistence of USUV neutralizing antibodies. However, the low USUV seroprevalence found in this survey and the fact that USUV RNA was not detected in any of the birds tested in spite of their origin, are consistent with the Italian and European scenarios [78,81].

## 5. Conclusions

In conclusion, we provided a descriptive assessment of potential flavivirus dispersal through migratory birds crossing Italy. The results on WNV obtained by our study do not exclude the “Migrant Bird as Introductory Host” hypothesis [82] and what was proposed by most of the studies of the last decade indicating infected long-distance migratory birds as spreaders of viruses from Africa to Europe. The low prevalence of WNV positive birds found in this survey, although not excluding, definitely indicates that long distance migratory birds played a marginal role in introducing new WNV strains from Africa as well as from Central and Northern European countries. Although dated, these data are in line with that shown by recent published data on the genomic evolution of WNV in Italy and Europe in which it appears clear that the WNV circulation in Italy and Europe is mainly determined by the inner circulation of local clusters and, just to a lesser extent, by external influxes [61,83]. Even though the virus was detected in an extremely low percentage of animals, we can assume a high real potential of WNV spread due to the movements of millions of birds of different orders every year originating from huge and extremely variable ranges and habitats where this virus is endemic.

Conversely, the results of this survey seem to indicate that the probability of new introductions of USUV to Italy through migratory birds is very low. This conclusion supports the findings of a recent study on the evolutionary processes and spatial spread of USUV strains circulating in the European context and in Italy in which the European origin of the four different lineages circulating in Italy was confirmed [84].

Through our survey it was possible to identify several wild species as potential amplifier hosts which are hardly detectable by passive surveillance either in urban or rural areas. Most of them, in fact, are strictly associated to different wild habitats which are usually not investigated, and the mortality rate is often very low and clinical signs are rarely reported. Furthermore, results of this work show how ringing activities can contribute to providing relevant information on status of bird-related diseases from a wide range of species. In Italy, as in Europe, a widespread network of hundreds of ringing sites operating through standardized protocols for monitoring projects could represent a new connection between the study of bird movements and pathogen circulation, within the perspective of a more extensive and active surveillance of zoonotic viruses.

## Figures and Tables

**Table 1:** WNV and USUV serological positives.

Year	Sampling Season	Order	Species	Migratory Strategy ^1^	N. Birds per Sample	N. Positive Samples									Total Positive Samples
						WNV ND50 ^2^						USUV ND50			
						1:10	1:20	1:40	1:80	1:160	1:320	1:10	1:20	1:40	
2013	Spring	Bucerotiformes	Hoopoe (*Upupa epops*)	L	1		1								1
					1								1		1
					1	2									2
		Caprimulgiformes	European Nightjar (*Caprimulgus europaeus*)	L	2		1								1
		Charadriiformes	Ruff (*Philomachus pugnax*)	L	1								1		1
			Wood Sandpiper (*Tringa glareola*)	L	2			1							1
					3		1								1
		Columbiformes	European Turtle Dove (*Streptopelia turtur*)	L	1	4	5	2	1						13
		Galliformes	Common Quail (*Coturnix coturnix*)	L	1	1	1								3
		Passeriformes	Common Whitethroat (*Sylvia communis*)	L	8	4									4
					9		1							1	2
			Eurasian Golden Oriole (*Oriolus oriolus*)	L	1	3	1	2							6
					2	1									1
			Garden Warbler (*Sylvia borin*)	L	8		1								1
					9					1					1
			Whinchat (*Saxicola rubetra*)	L	8	1									1
		Strigiformes	Eurasian Scops-Owl (*Otus scops*)	L	1			1							1
	Autumn	Charadriiformes	Redshank (*Tringa totanus*)	L	1		1								1
		Coraciiformes	European Bee Eater (*Merops apiaster*)	L	1				1	1					2
		Galliformes	Common Quail (*Coturnix coturnix*)	L	1	1									1
		Gruiformes	Spotted Crake (*Porzana porzana*)	L	1	3									3
		Passeriformes	Song Thrush (*Turdus philomelos*)	P	2								1		1
2014	Spring	Accipitriformes	Eurasian Sparrowhawk (*Accipiter nisus*)	P	1				1						1
			Marsh Harrier (*Circus aeruginosus*)	P	1	1									1
		Bucerotiformes	Hoopoe (*Upupa epops*)	L	1	2	1								3
		Columbiformes	European Turtle Dove (*Streptopelia turtur*)	L	1	5	5				1				11
		Passeriformes	Common Whitethroat (*Sylvia communis*)	L	6		1								1
					8	1									1
			Eurasian Golden Oriole (*Oriolus oriolus*)	L	2	1									1
					1	1		1	1		1				4
					2	3									3
		Strigiformes	Eurasian Scops-Owl (*Otus scops*)	L	1			1							1
	Autumn	Passeriformes	Hawfinch (*Coccothraustes coccothraustes*)	S	3		2								2
					3	1									1

Note: ^1^ S = short-distance migrant, L = long-distance migrant, P = partial migrant; ^2^ ND50 = (50% neutralization dose: concentration of antibodies that reduced the number of infected cells by 50%).

## Data Availability

The data presented in this study are available within the present manuscript and in supplementary material available at https://www.mdpi.com/article/10.3390/v14020416/s1.

## Supplementary Materials

The following supporting information can be downloaded at: https://www.mdpi.com/article/10.3390/v14020416/s1, Table S1: Details of sera collected for each bird species among years and seasons; Table S2: Number of organs (brain and kidneys) collected by different bird species found dead in Ventotene Island.
