# Supplementary material for: West Nile and Usutu Virus Introduction via Migratory Birds: A Retrospective Analysis in Italy
Source: Viruses. 2022 Feb 17;14(2):416. doi: 10.3390/v14020416 (PMC8880244; doi:10.3390/v14020416)
Supplement: Supplementary file 1 [file viruses-14-00416-s001.zip › Table S1.pdf]

Table S1. Details of sera collected for each bird species among years and seasons

Order	Species	Migratory Strategy[1]	Collection site[2]	Serum samples (n. individuals)					
				2012	2013		2014		Total
				Fall	Spring	Fall	Spring	Fall	
Anseriformes	Garganey (*Anas querquedula*)	L	M		1(1)				1(1)
Galliformes	Common Quail (*Coturnix coturnix*)	L	AL, BM, M, VI, ZI		142(159)	5(5)	1(1)		148(165)
Accipitriformes	Marsh Harrier (*Circus aeroginosus)*	P	VI			1(1)	1(1)		2(2)
	Eurasian Sparrowhawk (*Accipiter nisus*)	P	VI				1(1)		1(1)
Falconiformes	Kestrel (*Falco tinnunculus*)	P	VI				2(2)		2(2)
Gruiformes	Water Rail (*Rallus aquaticus*)	P	AL			48(48)			48(48)
	Spotted Crake (*Porzana porzana*)	L	AL			28(28)			28(28)
	Common Moorhen (*Gallinula chloropus*)	P	AL			2(2)			2(2)
Charadriiformes	Little Ringed Plover (*Charadrius dubius*)	L	CS			1(1)			1(1)
	Ringed Plover (*Charadrius hiaticula*)	S	CS	8(8)		10(11)			18(19)
	Grey Plover (*Pluvialis squatarola*)	L	CS	1(1)					1(1)
	Dunlin (*Calidris alpina*)	S	CS	2(2)		9(16)			11(18)
	Ruddy Turnstone (*Arenaria interpres*)	L	CS, O	2(2)					2(2)
	Curlew Sandpiper (*Calidris ferruginea*)	L	CS	9(9)		2(4)			11(13)
	Wood Sandpiper (*Tringa glareola*)	L	CS, M	15(15)	37(100)	32(56)			84(171)
	Green Sandpiper (*Tringa ochropus*)	L	CS	2(2)		1(2)			3(4)
	Common Sandpiper (*Actitis hypoleucos*)	L	CS, O	9(9)		5(9)			14(18)
	Redshank (*Tringa totanus*)	L	CS			15(19)			15(19)
	Spotted Redshank (*Tringa erythropus*)	L	CS	4(4)					4(4)
	Common Greenshank (*Tringa nebularia*)	L	CS, O	3(3)		3(3)			6(6)
	Marsh Sandpiper (*Tringa stagnatilis*)	L	CS			1(1)			1(1)
	Ruff (*Philomachus pugnax*)	L	M		12(12)				12(12)
	Black-headed Gull (*Chroicocephalus ridibundus*)	S	CS	2(2)					2(2)
	Mediterranean Gull (*Ichthyaetus melanocephalus*)	R	CS	4(4)					4(4)
	Sandwich Tern (*Thalasseus sandvicensis*)	L	CS	20(20)					20(20)
	Common Tern (*Sterna hirundo*)	L	CS	11(11)					11(11)
	Black Tern (*Chlidonias niger*)	L	CS	2(2)					2(2)
Columbiformes	European Turtle Dove (*Streptopelia turtur*)	L	VI, ZI, BM		92(99)		81(81)		173(180)
Cuculiformes	Common Cuckoo (*Cuculus canorus*)	L	VI		1(1)		5(5)		6(6)
Strigiformes	Boreal Owl (*Aegolius funereus*)	R, Irr	BC					1(1)	1(1)
	Eurasian Scops-Owl (*Otus scops*)	L	VI, ZI, BM, AL		6(8)	1(1)	7(7)		14(16)
Caprimulgiformes	European Nightjar (*Camprimulgus europaeus*)	L	VI, ZI		53(64)		11(11)		64(75)
Bucerotiformes	Hoopoe (*Upupa epops*)	L	VI, ZI, BM		16(25)		23(23)		39(48)
Coraciiformes	European Bee Eater (*Merops apiaster*)	L	VI, ZI, BM, AL		28(64)	12(21)	17(39)		57(124)
Piciformes	Wryneck (*Jynx torquata*)	L	VI				6(15)		6(15)
Passeriformes	Barn Swallow (*Hirundo rustica*)	L	ML, M		9(73)			60(416)	69(489)

Common Nightingale (*Luscinia megarhynchos*)	L	VI, ZI		4(29)		4(21)		8(50)
Common Redstart (*Phoenicurus phoenicurus)*	L	VI, ZI		18(140)		11(69)		29(209)
Northern Wheatear (*Oenanthe oenanthe*)	L	VI				6(26)		6(26)
Whinchat (*Saxicola rubetra*)	L	ZI		13(102)				13(102)
Song Thrush (*Turdus philomelos*)	P	BC, BAM, VI			21(45)	1(1)	51(116)	73(162)
Mistle Thrush (*Turdus viscivorus*)	P	BC, BAM			2(2)		4(4)	6(6)
Blackbird (*Turdus merula*)	P	BC, BAM			8(8)		37(38)	45(46)
Ring Ouzel (*Turdus torquatus*)	P	BC					3(3)	3(3)
Garden Warbler (*Sylvia borin*)	L	VI, ZI, C		32(255)		10(64)	1(10)	43(329)
Common Whitethroat (*Sylvia communis*)	L	VI		33(280)		5(30)		38(310)
Icterine Warbler (*Hippolais icterina*)	L	VI		9(80)		5(41)		14(121)
Woodchat Shrike (*Lanius senator*)	L	VI				7(16)		7(16)
Nutcracker (*Nucifraga caryocatactes*)	R, Irr	BC			1(1)			1(1)
Eurasian Golden Oriole (*Oriolus oriolus*)	L	ZI, VI		58(71)		55(87)		113(158)
Crossbill (*Loxia curvirostra*)	S	BC					12(32)	12(32)
Hawfinch (*Coccothraustes coccothraustes*)	S	BC					41(86)	41(86)
Total			94(94)	564(1563)	209(294)	259(541)	209(696)	1335(3188)

Note: [1] L=long distance migrant, S=short distance migrant, P=partial migrant, Irr=irregular migrant, R=resident;
[2] AL= Alfieri Lake, BAM=Barro Mountain, BC=Bocca di Caset, BM=Brisighella Mountain, C=Campotto, CS=Comacchio Salina, M=Mirandola, ML=Matese Lake, O=Ortazzo, VI=Ventotene Island, ZI=Zannone Island.
