# Supplementary material for: West Nile and Usutu Virus Introduction via Migratory Birds: A Retrospective Analysis in Italy
Source: Viruses. 2022 Feb 17;14(2):416. doi: 10.3390/v14020416 (PMC8880244; doi:10.3390/v14020416)
Supplement: Supplementary file 1 [file viruses-14-00416-s001.zip › Table S2.pdf]

Table S2. Number of organs (brain and kidneys) collected by different bird species found dead in Ventotene Island

Order	Species	Migratory Strategy[1]	Year			
			2013		2014	
			Brain	Kidneys	Brain	Kidneys
Charadriiformes	Yellow-legged Gull (*Larus michahellis*)	R			1	1
Galliformes	Common Quail (*Coturnix coturnix*)	L	1	1	3	3
Cuculiformes	Common Cuckoo (*Cuculus canorus*)	L	1	1		
Coraciiformes	European Bee Eater (*Merops apiaster*)	L	1	1		
Passeriformes	Barn Swallow (*Hirundo rustica*)	L	1	1		
	Meadow Pipit (*Anthus pratensis*)	S	1	1		
	Tree Pipit (*Anthus trivialis*)	L	7	7	2	2
	Robin (*Erithacus rubecola*)	S	2	2	4	4
	Nightingale (*Luscinia megarhyncos*)	L	8	8	2	2
	Common Redstart (*Phoenicurus phoenicurus*)	L	6	6	5	5
	Whinchat (*Saxicola rubetra*)	L	16	16	2	1
	Song Thrush (*Turdus philomelos*)	P			1	2
	Garden Warbler (*Sylvia borin*)	L	7	6	20	21
	Blackcap (*Sylvia atricapilla*)	S	1	1		
	Common Whitethroat (*Sylvia communis*)	L	11	11	7	7
	Sardinian Warbler (*Sylvia melanocephala*)	P	1	1		
	Subalpine Warbler (*Sylvia cantillans*)	L	10	11	7	6
	Reed Warbler (*Acrocephalus scirpaceus*)	L			1	1
	Great Reed Warbler (*Acrocephalus arundinaceus*)	L	1	1		
	Icterine Warbler (*Hippolais icterina*)	L	6	8	8	9
	Willow Warbler (*Phylloscopus trochilus*)	L	7	8	9	9
	Wood Warbler (*Phylloscopus sibilatrix*)	L	27	28	6	7
	Western Bonelli's Warbler (*Phylloscopus bonelli*)	L		1		
	Common Chiffchaff (*Phylloscopus collybita*)	S	3	4	4	4
	Spotted Flycatcher (*Muscicapa striata*)	L	8	8	2	3
	Pied Flycatcher (*Ficedula hypoleuca*)	L	31	30	5	6
	Collared Flycatcher (*Ficedula albicollis*)	L	8	8	1	1
	Ficedula sp.	L	1	1		
	Eurasian Golden Oriole (*Oriolus oriolus*)	L	1	1	1	1
	Serin (*Serinus serinus*)	P	2	2		
Total			169	174	91	95

Note: [1] S=short distance migrant, L=long distance migrant, P=partial migrant, R=resident.
